# Supplementary material for: Simulated Microgravity Alters P-Glycoprotein Efflux Function and Expression via the Wnt/β-Catenin Signaling Pathway in Rat Intestine and Brain
Source: Int J Mol Sci. 2023 Mar 12;24(6):5438. doi: 10.3390/ijms24065438 (PMC10049079; doi:10.3390/ijms24065438)
Supplement: Supplementary file 1 [file ijms-24-05438-s001.zip › Figure_S2.pdf]

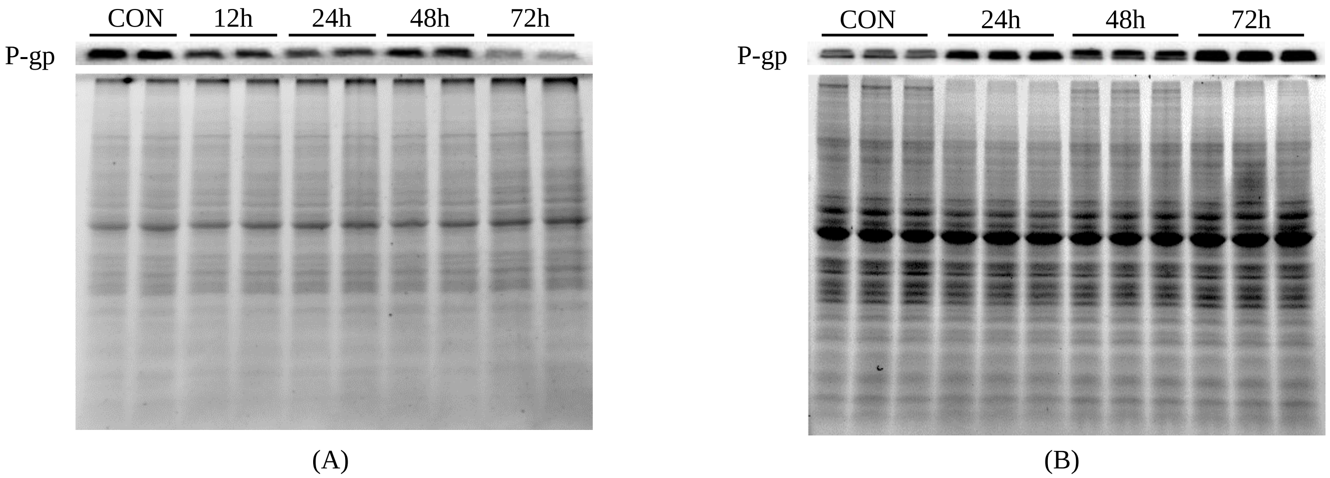

Supplementary Figure S2 The protein bands and total proteins gels of Western-Blot corresponding to Figure 2 and Figure 4. (**A**) Western-Blot bands of P-gp and total proteins gels in Caco-2 cells. (**B**) Western-Blot bands of P-gp and total proteins gels in hCMEC/D3 cells.
