# Supplementary material for: Simulated Microgravity Alters P-Glycoprotein Efflux Function and Expression via the Wnt/β-Catenin Signaling Pathway in Rat Intestine and Brain
Source: Int J Mol Sci. 2023 Mar 12;24(6):5438. doi: 10.3390/ijms24065438 (PMC10049079; doi:10.3390/ijms24065438)
Supplement: Supplementary file 1 [file ijms-24-05438-s001.zip › Figure_S3.pdf]

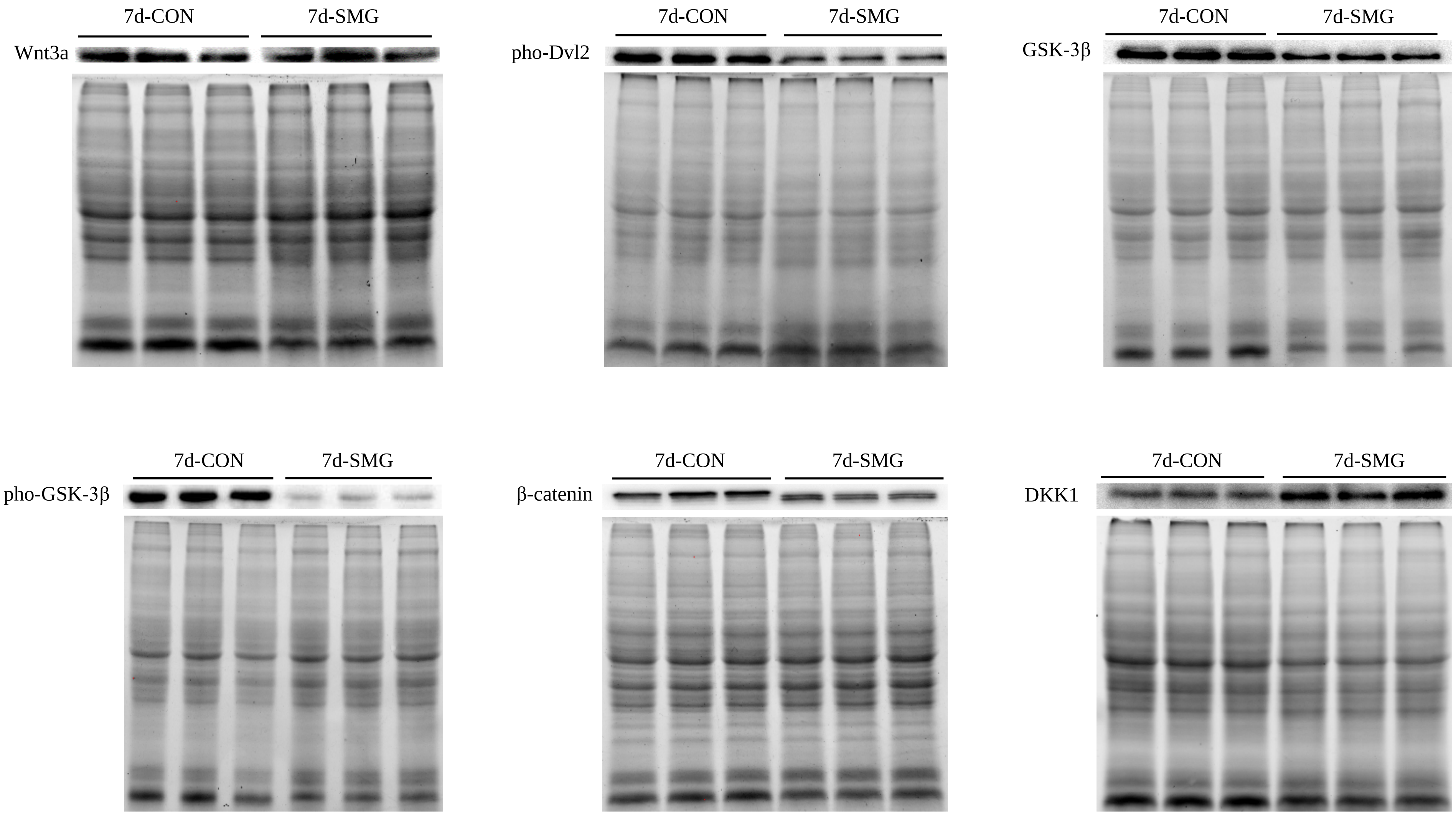

(A

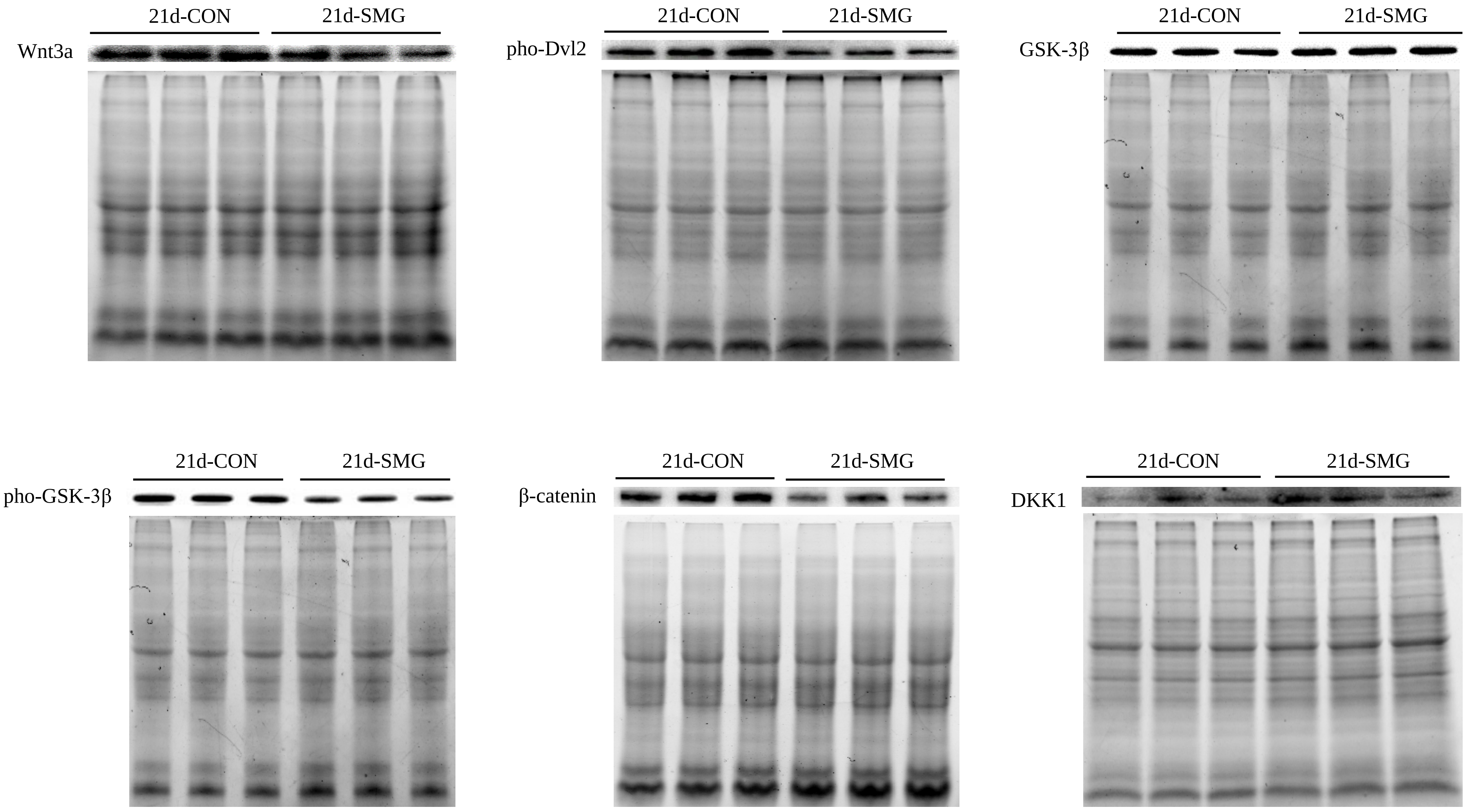

(B

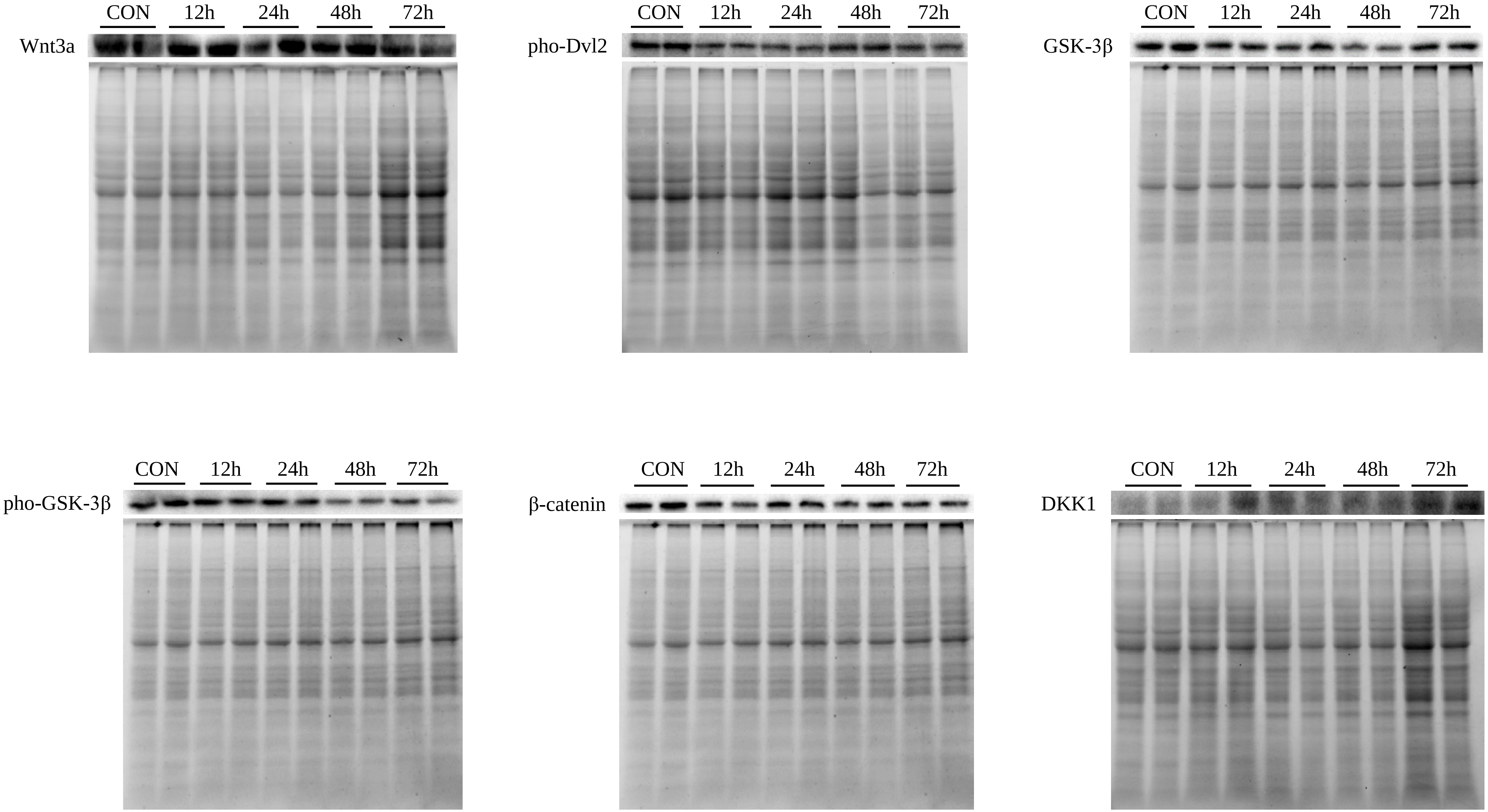
CON 12h 24h 48h 72h
Wnt3a
pho-Dvl2
GSK-3β
pho-GSK-3β
β-catenin
DKK1

(C)

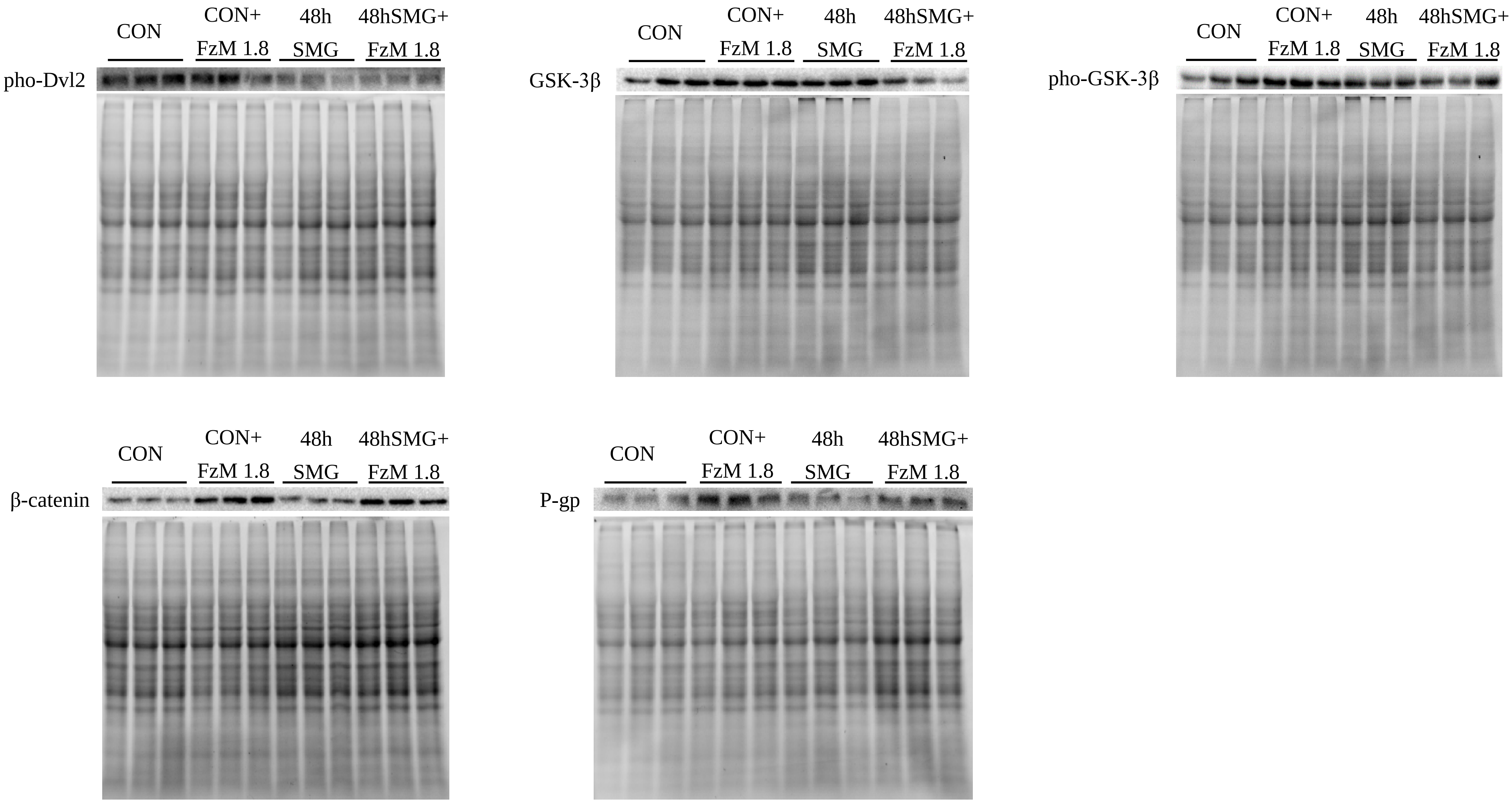

(D)

Supplementary Figure S3 The protein bands and total proteins gels of Western-Blot corresponding to Figure 5. (**A-B**) Western-blot bands of pho-Dvl2, GSK-3β, pho-GSK-3β, β-catenin, DKK1 and total proteins gels in rat ileum. (**C-D**) Western-blot bands of Wnt3a, pho-Dvl2, GSK-3β, pho-GSK-3β, β-catenin, P-gp and total proteins gels in Caco-2 cells.
