# Supplementary material for: Simulated Microgravity Alters P-Glycoprotein Efflux Function and Expression via the Wnt/β-Catenin Signaling Pathway in Rat Intestine and Brain
Source: Int J Mol Sci. 2023 Mar 12;24(6):5438. doi: 10.3390/ijms24065438 (PMC10049079; doi:10.3390/ijms24065438)
Supplement: Supplementary file 1 [file ijms-24-05438-s001.zip › Figure_S4.pdf]

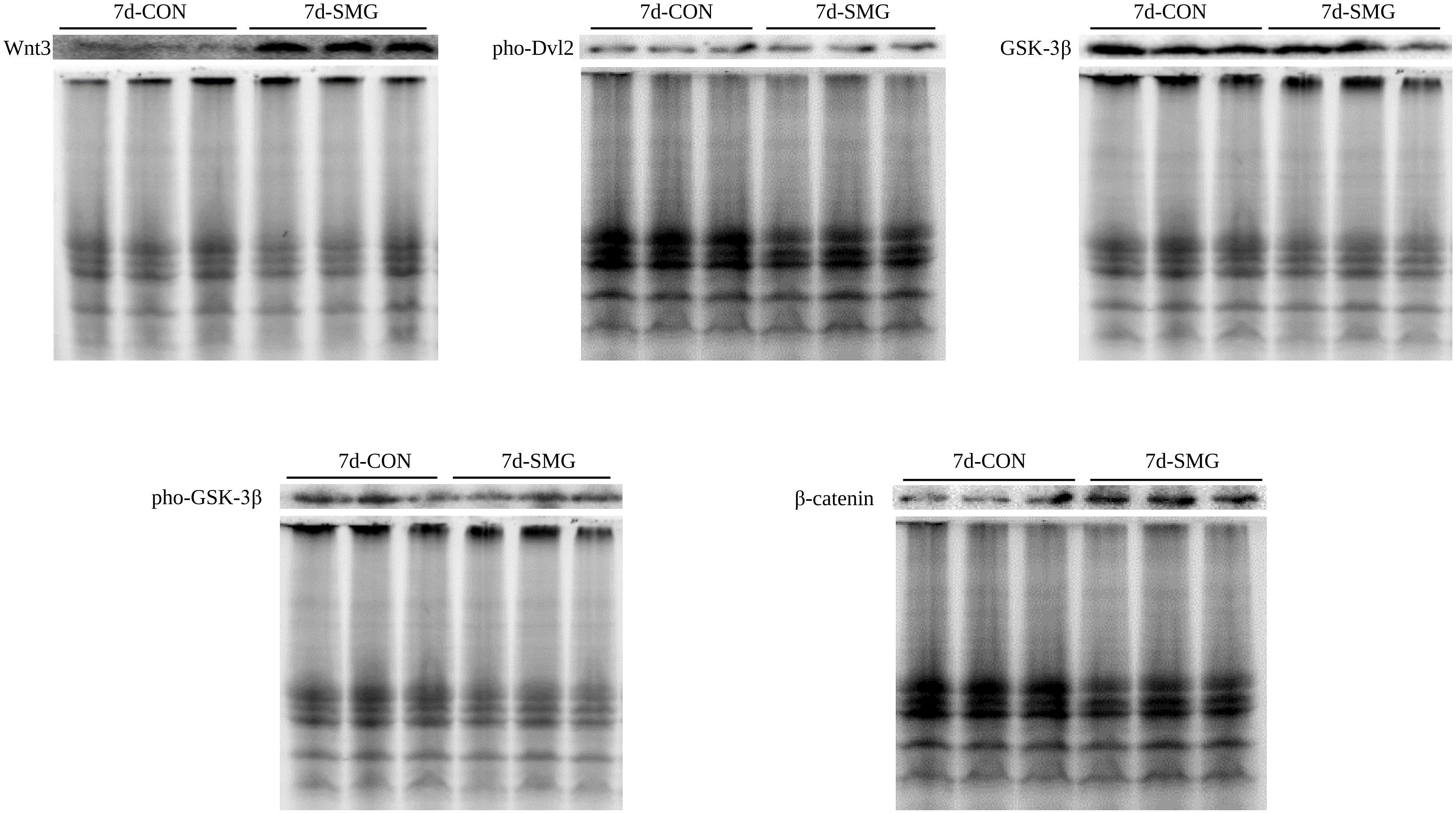
7d-CON
7d-SMG
Wnt3
7d-CON
7d-SMG
pho-Dvl2
7d-CON
7d-SMG
GSK-3β
7d-CON
7d-SMG
pho-GSK-3β
7d-CON
7d-SMG
β-catenin

(A

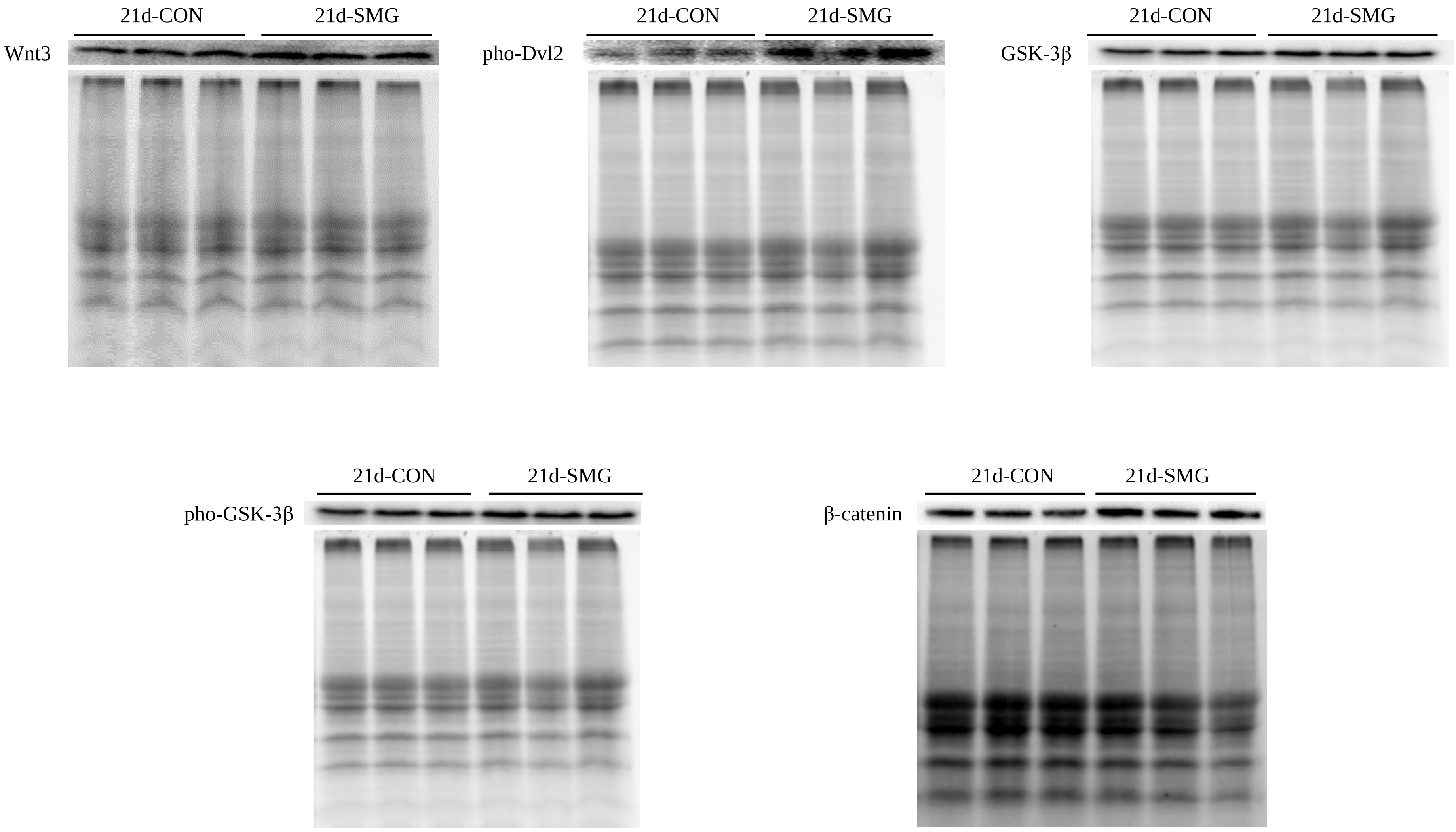
21d-CON
21d-SMG
Wnt3
21d-CON
21d-SMG
pho-Dvl2
21d-CON
21d-SMG
GSK-3β
21d-CON
21d-SMG
pho-GSK-3β
21d-CON
21d-SMG
β-catenin

(B)

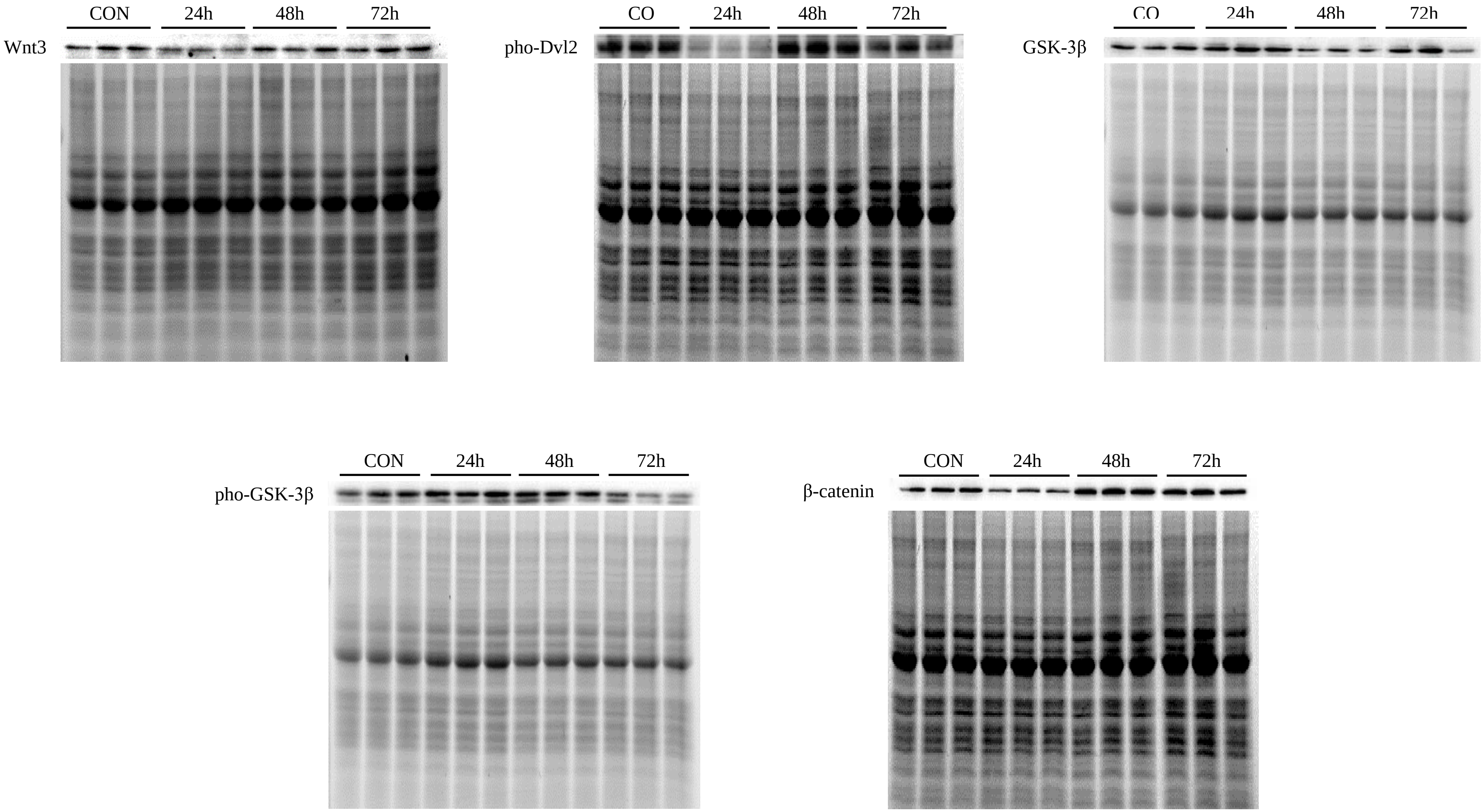
CON
24h
48h
72h
Wnt3
CO
24h
48h
72h
pho-Dvl2
CO
24h
48h
72h
GSK-3β
CON
24h
48h
72h
pho-GSK-3β
CON
24h
48h
72h
β-catenin

(C)

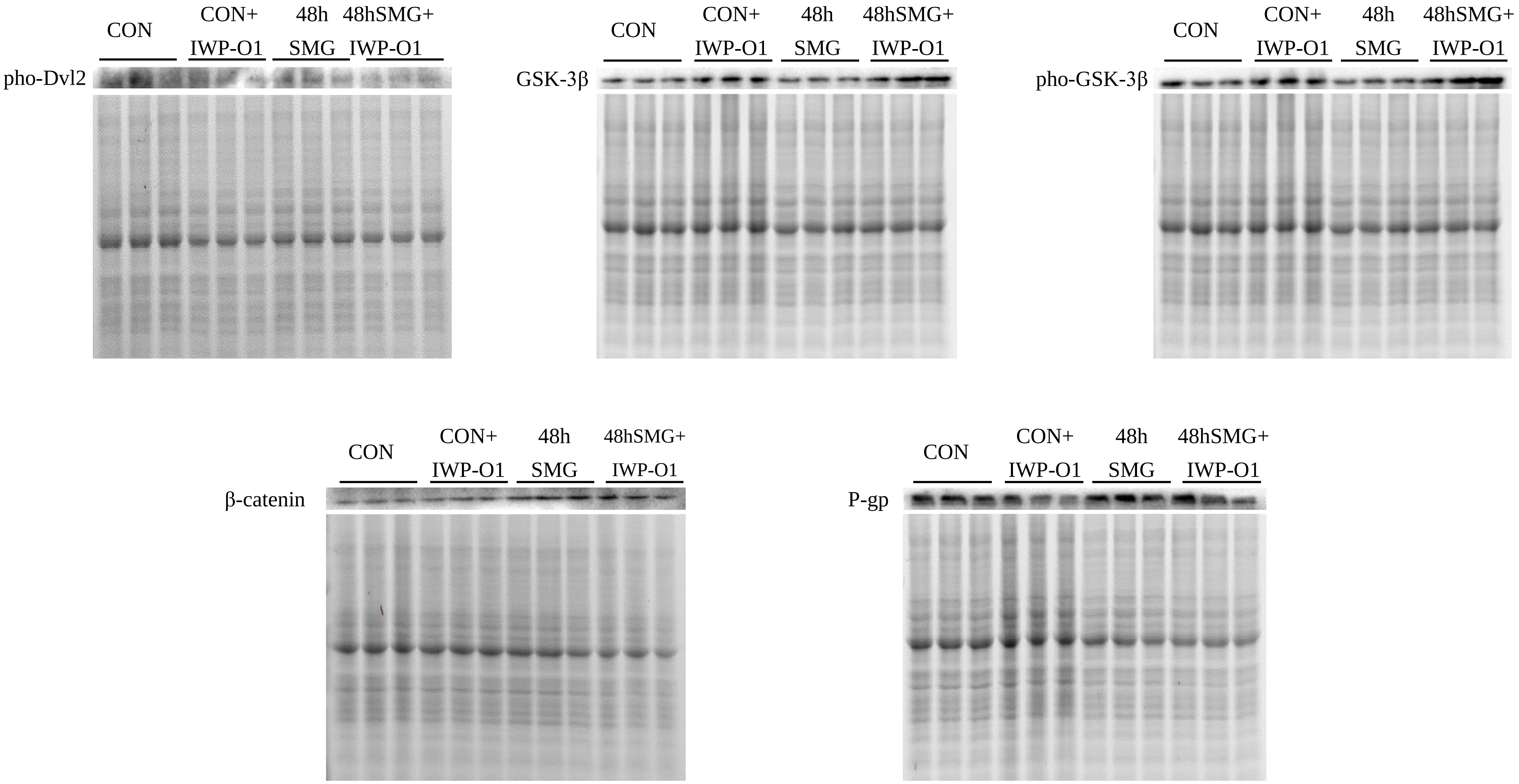

(D

Supplementary Figure S4 The protein bands and total proteins gels of Western-Blot corresponding to Figure 6. (A-B) Western-blot bands of Wnt3, pho-Dvl2, GSK-3β, pho-GSK-3β, β-catenin, DKK1 and total proteins gels in rat brain. (C-D) Western-blot bands of Wnt3, pho-Dvl2, GSK-3β, pho-GSK-3β, β-catenin, P-gp and total proteins gels in hCMEC/D3 cells.
