# Supplementary material for: Simulated Microgravity Alters P-Glycoprotein Efflux Function and Expression via the Wnt/β-Catenin Signaling Pathway in Rat Intestine and Brain
Source: Int J Mol Sci. 2023 Mar 12;24(6):5438. doi: 10.3390/ijms24065438 (PMC10049079; doi:10.3390/ijms24065438)
Supplement: Supplementary file 1 [file ijms-24-05438-s001.zip › Figure_S5.pdf]

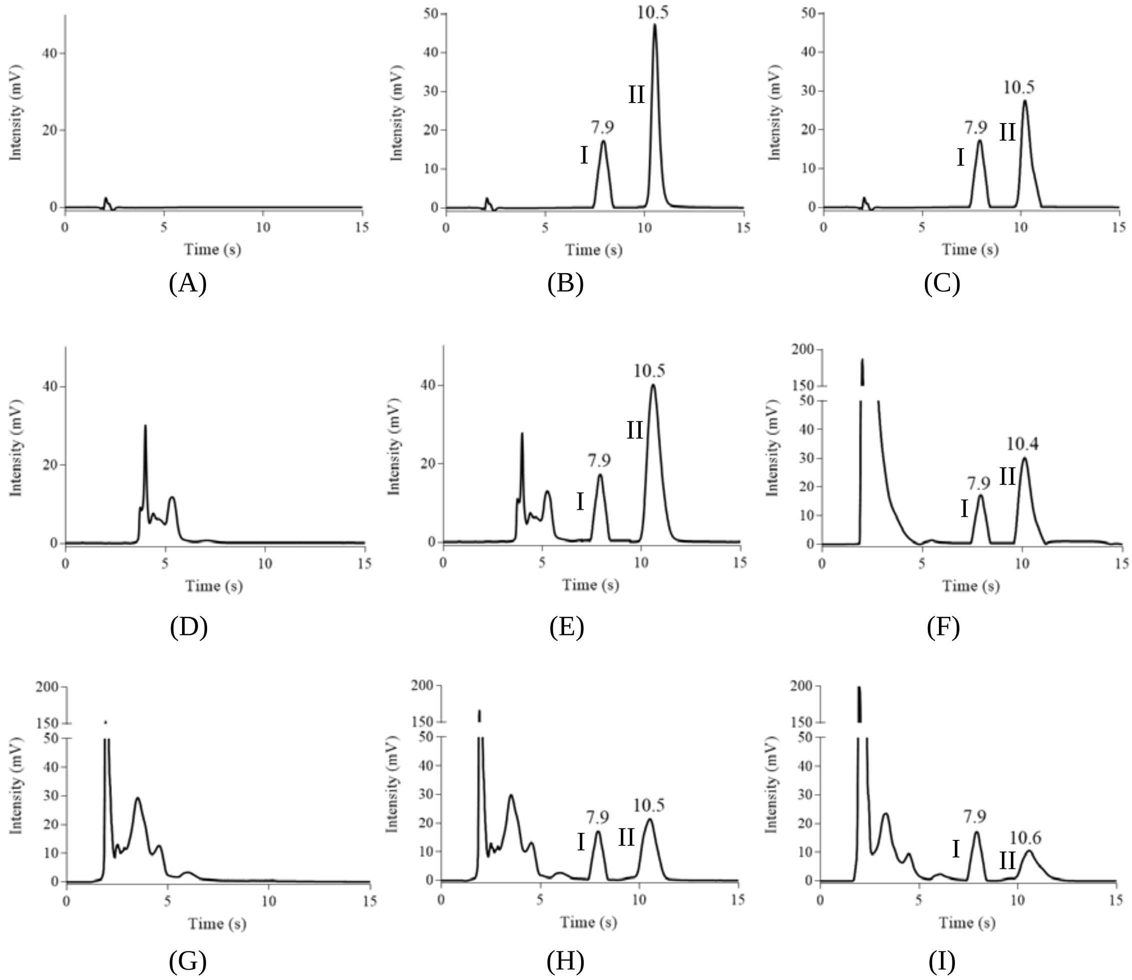

Supplementary Figure S5 Representative chromatograms. (**A**) Blank KR buffer. (**B**) Blank KR buffer-spiked with ferulic acid (100 μg/mL) as internal standard and paracetamol (40 μg/mL). (**C**) Actual intestinal perfusion collection fluid spiked with ferulic acid (100 μg/mL) as internal standard. (**D**) Blank rat plasma. (**E**) Blank rat plasma with ferulic acid (100 μg/mL) as internal standard and paracetamol (40 μg/mL). (**F**) Actual rat plasma samples spiked with ferulic acid (100 μg/mL) as internal standard. (**G**) Blank rat brain. (**H**) Blank rat brain spiked with ferulic acid (100 μg/mL) as internal standard and paracetamol (40 μg/mL). (**I**) Actual rat brain sample spiked with ferulic acid (100 μg/mL) as internal standard. Notes: I, internal standard ferulic acid, T_R 7.9 min; II, paracetamol, T_R 10.5 min.
